# Depression, sleep quality and condom use amongst Iranian people living with human immunodeficiency virus

**DOI:** 10.4102/sajhivmed.v21i1.1150

**Published:** 2020-12-15

**Authors:** Marzieh Mahboobi, Arezu Najafi, Amin Nakhostin-Ansari, Parvin Afsar Kazerooni, Matin Bazargani, Fatemeh Navaiian, Samaneh Akbarpour

**Affiliations:** 1 Department of Epidemiology and Biostatistics, School of Public Health, Isfahan University of Medical Sciences, Isfahan, Iran; 2 Occupational Sleep Research Center, Baharloo Hospital, Tehran University of Medical Sciences, Tehran, Iran; 3 Sports Medicine Research Center, Neuroscience Institute, Tehran University of Medical Sciences, Tehran, Iran; 4 Center for Communicable Disease Control, Ministry of Health and Medical Education, Tehran, Iran; 5 HIV Expert of Deputy of Health, Tehran University of Medical Sciences, Tehran, Iran; 6 HIV Expert of Deputy of Health, Shahid Beheshti University of Medical Sciences, Tehran, Iran

**Keywords:** Depression, condom use, HIV, Iran, sleep

## Abstract

**Background:**

Depression is a common mood disorder in people living with human immunodeficiency virus (PLWH) and is associated with risk-taking sexual behaviour.

**Objectives:**

This study examines depression, sleep quality and condom usage amongst PLWH in Tehran, Iran.

**Method:**

This cross-sectional study was undertaken between October and November 2019 on 298 PLWH referred to voluntary counselling and testing centres (VCTs) in Tehran. Participants provided information as per the following questionnaires: the Depression, Anxiety, Stress Scale (DASS), the Pittsburgh Sleep Quality Index Questionnaire and a demographic questionnaire that evaluated condom use.

**Results:**

A total of 298 PLWH, of whom 202 (67.8%) were men with a mean age of 39.81 years, were enrolled in the study. The DASS classified 57% with depression. Fewer than 20% of these used condoms regularly. The majority of depressed patients were men (68.6%) and 31.4% were women. The depressed patients were more likely *not* to use condoms than those who were not depressed (adjusted odds ratio [OR] = 6.5; 95% confidence interval [CI], 3.70–11.42). The adjusted OR for not using a condom amongst the depressed was 7.12 times greater (95% CI, 5.85–10.11) than in those without depression.

**Conclusion:**

Our findings suggest that depression is common amongst PLWH in Tehran and is associated with risk-taking sexual behaviour. Appropriate interventions are needed to address mental disorders in PLWH. It is recommended that patients be screened regularly for symptoms of depression and, where indicated, counselled and managed.

## Introduction

The World Health Organization (WHO) reports that at the end of 2018, approximately 38 million people were living with human immunodeficiency virus (HIV) globally.^1^ The general health of these patients has improved, and life expectancy and survival have increased.^2^ Nonetheless, social problems, negative attitudes and stigma remain and affect the mental health of people living with HIV (PLWH). Mental disorders are common in this group and include depression, mania, psychosis, anxiety, substance abuse and the risk of suicide.^3^

Multiple sexual partners and drug and alcohol abuse are more likely amongst depressed than non-depressed people.^4,5,6^ These behaviours are also associated with the acquisition of HIV.^7^ Depression has been linked to unreliable adherence to antiretroviral therapy (ART) and treatment failure. Indeed, risk-taking sexual behaviour, poor adherence to treatment and ART failure require the exclusion of depression as a cause.^8,9^ The correct and consistent use of condoms reduces HIV transmission and other sexually transmitted infections (STIs).^10,11,12^ Condom use is influenced by depression. In a study of 278 Chinese women at high risk of HIV infection, 62% had high levels of depression. Those with more severe depression were more likely not to use a condom or to misuse it.^13^

Although studies of condom use have been conducted in various countries, the generalisability of these to Iranian PLWH is uncertain in view of significant cultural, social and economic differences. Furthermore, Iranian studies of condom use in PLWH and other at-risk groups with mood disorders are few.^14,15,16^ Given the importance of condoms in preventing STIs, this study aimed to identify associations between depression, sleep quality and condom use in this cohort of PLWH.

## Materials and methods

This study was performed as a cross-sectional study on 298 PLWH referred to voluntary counselling and testing centres (VCTs) in Tehran, the capital of Iran, from October to November 2019. We evaluated depression, sleep quality and condom use in PLWH and assessed the presence of independent associations between these factors.

As Tehran is a large city, to ensure the random selection of VCTs from all regions of the city, it was divided into four geographical areas – north, south, east and west. After that, a list of these centres was prepared separately for each of the four regions. In each area, two VCTs were randomly selected, for a total of eight centres. Finally, the existing files in each centre were randomly selected, and people were invited to participate in the study.

A demographic questionnaire, the Depression Anxiety Stress Scale (DASS) and the Pittsburgh Sleep Quality Index Questionnaire (PSQI) were used for data collection. The DASS questionnaire is designed to address depression and anxiety overlap. This scale has two different versions. In the main version, which has 42 questions, each of the psychological structures (depression, anxiety and stress) is evaluated by 14 different questions, but in its abbreviated version, there are 21 questions, and each psychological structure is measured by seven questions. Each question can be answered by ‘never’, ‘sometimes’, ‘often’ or ‘almost always’. Various studies indicate that the DASS questionnaire provides valid assessments of the three structures of depression, anxiety and stress and can significantly differentiate between different contexts.^17^

In this study, the Persian translation of the abbreviated version of the questionnaire was used. The validity and reliability of the Persian version have been confirmed in several studies.^18,19^ To assess the quality of sleep in patients in the last month, the PSQI was used. This questionnaire has seven components: quality of sleep, sleep delay, sleep duration, sleep efficiency, sleep disorders, sleeping pill use and daily dysfunction. Many studies have confirmed the validity and reliability of this questionnaire.^20,21^ Each component can be scored from 0 to 3, and an overall score of 5 and above indicates inappropriate or poor quality of sleep.^22^ Background demographic data also collected included gender, age, education, marital status, body mass index (BMI), employment status, CD4 count, time since HIV diagnosis, route of transmission, concurrent hepatitis B and C and tuberculosis infections and duration of ART. Participants were asked about condom use during the past year. The response was divided into three parts: people who never used a condom, people who used it sometimes and people who always used a condom.

Our inclusion criteria were (1) confirmation of HIV infection by rapid and enzyme-linked immunosorbent assay HIV tests, (2) a history of sexual intercourse at least once over the past 12 months, (3) age 18 years or older and (4) informed consent to participate in the study. Participants whose condom use status was unclear (*n* = 8) were excluded from the sample. Other participants were included in the study.

Questions were asked in face-to-face, one-on-one interviews by eight trained interviewers (psychologists). The interviewers provided participants with an explanation of the study and its objectives and invited participants to consider enrolling. Participants were assured of confidentiality and an ‘opt-out option’ if they did not wish to continue with the study. Informed consent was obtained from participants prior to the study.

### Statistical analysis

For quantitative data, mean and standard deviation (SD) were used; frequency and percentage were reported for the qualitative variables. One-way analysis of variance and *t*-test were used to evaluate the differences between depressed and non-depressed individuals in terms of age, BMI, duration since the HIV diagnosis, CD4 count and duration of ART. We used a chi-square test to evaluate differences between depressed and non-depressed individuals in terms of gender, educational level, marital status, condom use status, sleep quality, anxiety, stress, route of HIV transmission and coinfections.

The multinomial logistic regression method was used to determine the relationship between depression, age, sex, marital status, duration since the diagnosis of HIV and the use of condoms, and people who always used condoms were defined as a reference. All analyses were performed with Stata software, and a *p*-value of less than 0.05 was considered significant.

### Ethical consideration

This study was approved by the ethical committee of Tehran University of Medical Sciences (ethics approval number, IR.TUMS.VCR.REC.1398.312).

## Results

A total of 298 HIV-positive patients, including 96 women (32.2%) and 202 men (67.8%), participated in the study. The average age of participants was 39.81 (SD = 9.67) years. More than half were married. About half of the participants were illiterate or had less than 12 years of formal education, namely, ‘under diploma’ status. The mean BMI of the participants was 24.5 kg/m^2^. In all, 30% of participants never used condoms, 32% sometimes used condoms and 37.1% always used condoms. The demographics of the participants, their condom use status and their sleep quality are tabulated in Table 1.

**TABLE 1a:** Demographic characteristics of the depressed and non-depressed participants.

Variable	Total (*n* = 298)		Depressed (*n* = 169)		Non-depressed (*n* = 129)		*p*
	Mean	SD	Mean	SD	Mean	SD	
Age, years (mean ± SD)	39.81	9.67	39.98	10.19	39.59	8.98	0.734
BMI (kg/m^2^)	24.78	4.16	25.73	4.34	24.06	3.88	0.001

**TABLE 1b:** Demographic characteristics of the depressed and non-depressed participants.

Variable	*n*	%	*n*	%	*n*	%	*p*
**Sex**							< 0.0001
Female	96	32.2	53	55.2	43	44.8	-
Male	202	67.8	116	57.4	86	42.6	-
**Marital status**							0.191
Married (committed)	155	52	79	50.9	76	49.1	-
Single†	143	48	90	62.9	53	37.1	-
**Education**							< 0.0001
Under diploma	140	47	86	50.9	54	41.9	-
Diploma	104	34.9	62	59.6	42	40.4	-
Upper diploma	54	18.1	21	38.8	33	61.2	-
**Current employment status**							0.0100
Employed	217	72.8	111	51.1	106	48.9	-
Unemployed	81	27.2	58	71.6	23	28.4	-
**Condom use status**							< 0.0001
Never use condom	91	30.5	71	78	20	22	-
Sometimes use condom (inconsistent)	96	32.2	66	68.7	30	31.3	-
Always use condom (consistent)	111	37.2	32	28.8	79	71.2	-
**Poor sleep quality (PSQI, > 5)**	210	70.4	139	82.2	71	55	< 0.0001
**Anxiety (> 6)**	154	51.7	127	75.1	27	20.9	< 0.0001
**Stress (> 10)**	204	68.5	154	91.1	50	38.8	< 0.0001

BMI, body mass index; PSQI, Pittsburgh Sleep Quality Index.

† , Never married or divorced.

The overall prevalence of depression was 56.7%. Almost 70% of those with depression were men, and more than half of the depressed patients were unemployed and had not completed formal education (*p* < 0.05). The depressed group had a significantly higher BMI than the non-depressed group. Amongst 169 depressed individuals, only 32 (18.9%) always used condoms, whilst 71 (42%) never used condoms. Almost 70% of the participants had a PSQI of > 5, which means that they had poor sleep quality. Eighty-two per cent (*n* = 139) of PLWH with depression suffered from poor sleep quality. Similarly, 75% and 91% of depressed people had symptoms of anxiety and stress, respectively. Table 2 shows the HIV infection status in depressed and non-depressed participants. The main route of HIV transmission to participants was unsafe drug injection. About 24% of people with depression were co-infected with HIV and hepatitis C.

**TABLE 2a:** Clinical status of depressed and non-depressed participants.

Variable	Total (*n* = 298)		Depressed (*n* = 169)		Non-depressed (*n* = 129)		*p*
	Mean	SD	Mean	SD	Mean	SD	
Time since HIV diagnosis, months	62.32	49.21	56.52	43.76	67.51	54.90	0.056
CD4 count	577.03	301.56	586.98	321.23	573.24	277.50	0.698
Duration of ART, months (med, IQR)	46.19	36.61	42.44	32.17	50.17	41.87	0.073

**TABLE 2b:** Clinical status of depressed and non-depressed participants.

Variable	*n*	%	*n*	%	*n*	%	*p*
**CD4 count**							0.408
< 500	129	43.3	76	45	53	41.1	-
≥ 500	169	56.7	93	55	76	58.9	-
**Route of HIV acquisition**							0.087
Sexual contact	120	40.3	68	40.2	52	40.3	-
Injection drug use	167	56	98	58	69	53.5	-
Blood products	4	1.3	1	0.6	3	2.3	-
Unknown	7	2.3	2	1.2	5	3.9	-
**HIV/HBV coinfection**	9	3	8	4.7	1	0.8	0.373
**HIV/HCV coinfection**	62	20.8	41	24.3	21	16.3	0.376
**HIV/TB coinfection**	26	8.7	13	7.7	13	10.1	0.693

HIV, human immunodeficiency virus; ART, antiretroviral therapy; HBV, hepatitis B virus; HCV, hepatitis C virus; TB, tuberculosis.

Table 3 depicts the results of logistic regression, indicating that the odds of not using a condom in people with depression are higher compared to those who do not have depression (odd ration [OR], 6.50; 95% confidence interval [CI], 3.70–11.42).

**TABLE 3:** Results of the unadjusted and adjusted logistic regression to assess the relation between depression and not using condoms amongst people living with human immunodeficiency virus.

Variable	OR	95% CI	*p*
**Model 1**			
Crude adjusted value	6.76	4.00–9.41	< 0.0001
**Model 2**			
Age and sex adjusted	6.66	3.43–7.66	< 0.0001
**Model 3†**			
Multivariate adjusted	6.50	3.70–11.42	< 0.0001

OR, odds ratio; CI, confidence interval; HIV, human immunodeficiency virus.

Not using a condom was defined as an outcome in the logistics model.

† , Model 3 was adjusted for age, sex, education, time since HIV infection and marital status.

According to Table 4, in multinomial logistic regression, people with depression tended to be about 7.12 and 5.01 times more likely to not use or occasionally use condoms, respectively, compared to those who were not depressed.

**TABLE 4:** Results of unadjusted and adjusted multinomial logistic regression to assess the relation between depression and not continuously using condoms amongst people living with human immunodeficiency virus.

Variable	No vs. consistent condom use (*n* = 91/111)			Inconsistent vs. consistent condom use (*n* = 96/111)		
	OR	95% CI	*p*	OR	95% CI	*p*
**Model 1**						
Crude adjusted value	7.76	5.60–11.16	< 0.0001	5.54	2.99–7.84	< 0.0001
**Model 2**						
Age and sex adjusted	7.06	5.82–19.76	< 0.0001	5.83	3.17–8.71	< 0.0001
**Model 3†**						
Multivariate adjusted	7.12	5.85–10.11	< 0.0001	5.01	3.69–8.33	< 0.0001

OR, odds ratio; CI, confidence interval; HIV, human immunodeficiency virus.

Consistent condom used was defined as condoms always used. Inconsistent condom used was defined as condoms sometimes used.

† , Model 3 was adjusted for age, sex, education, time since HIV infection and marital status.

## Discussion

Whilst many studies have looked at the prevalence of condom use in different populations, including PLWH, research on condom use in people with mood disorders, including depression, is very limited. Considering the importance of using condoms in PLWH in the prevention of the transmission of infection to others, as well as the importance of the infected patients’ health, this study evaluated the relationship between depression and condom use in PLWH in Tehran. The results of the present study showed that less than 40% of the participants in the study used condoms continuously, and almost 30% did not use condoms at all. A study conducted by Lotfi et al. on 121 HIV-positive people in Karaj reported that about 60% of participants had not used condoms during sexual intercourse in the preceding 3 months.^14^ Similar studies in London and Uganda reported a prevalence of condom use of 73% and 65% in PLWH, respectively.^23,24^ The differences observed between Iranian and non-Iranian PLWH can be a result of cultural differences, lower levels of education and lack of sufficient knowledge about the importance of continuous and correct use of condoms amongst Iranian PLWH, carelessness^25^ and lack of continuous access to condoms. Depression in PLWH is associated with unreliable condom use, with drug and alcohol misuse, poor adherence to treatment, detectable viral load on ART, and high-risk sexual behaviour.^8,9^ Considering the high prevalence of depression amongst Iranian PLWH and the sequelae of depression, there is a need for interventions to address the depression problem amongst Iranian PLWH. More than half of those living with HIV in the present study had depression. Only 19% reported regular condom use. Wagner et al. evaluated the effects of ART and depression on condom use in Ugandan PLWH. They found that over time, depression is accompanied in PLWH by a reduction in the persistent use of condoms.^24^ An American study confirmed a relationship between depression and the failure to use condoms in PLWH with uninfected partners.^26^

In our study, depression was more common in men than in women. Shadloo et al. evaluated the prevalence of psychological issues amongst Iranian PLWH: mood disorders, including major depressive disorder, were more prevalent in males than females (34.5% vs. 28.8%, respectively).^27^ Pasdar et al. evaluated the association between dietary intake and depression in PLWH in Iran and found depression to be more common amongst Iranian males living with HIV than females.^28^ In a systematic review and meta-analysis, Rezaei et al. evaluated depression globally amongst PLWH and noted a prevalence of 8% amongst males. This was significantly higher compared to females.^29^ Men clearly are at significant risk of depression, and test and treat programs that care for PLWH need to address the issue proactively.

It also appears that depression is more common in patients with lower educational levels, those who are unemployed and those who are overweight (BMI, > 25). Having a job and sufficient income is one of the most important social factors affecting health and has a significant impact on the mental state of individuals. Unemployment or low-income work promote the cycle of poverty and limit access to healthcare.^30,31^

Eighty-two per cent of depressed PLWH had poor sleep quality. Poor sleep quality can lead to daily drowsiness, mood swings, decreased quality of social interaction, exacerbation of depression, anxiety and increased high-risk behaviours.^32^ It is also a risk factor for mood disorders.^33^ Downing et al. found that good sleep quality is associated with less depression and anxiety and more self-efficient condom use in PLWH.^34^

Lack of questions about the reasons for not using condoms in patients is one limitation of our study, as identifying the barriers to protective behaviour could play an essential role in planning strategies to remove these barriers. Another limitation is the use of face-to-face interviews for data collecting, which was challenging in some cases, given Iran’s cultural, religious and social context. This may have led to various biases (interviewer and reminder bias or bias of social desirability). Thirdly, it was a cross-sectional study, and we could not evaluate the transmission of the virus to sexual partners in the depressed and non-depressed patients. Fourthly, we did not assess the patients’ viral load, which can also affect the transmission of the virus to patients’ sexual partners. Fifthly, we used a questionnaire to see whether the patients were depressed or not, which is another limitation of our study as confirmation of depression by a psychologist could give a more accurate assessment.

## Conclusion

In this study, we evaluated the prevalence of depression in PLWH and its associations with sleep quality and condom use. The prevalence of depression in Iranian PLWH is relatively high, and interventions are needed to address this problem. Furthermore, depressed PLWH have worse sleep quality and use condoms less frequently compared to non-depressed PLWH. Interventions to improve the sleep quality and mood of PLWH may be valuable considering their associations with condom use.
